# School Nurses’ perspectives on the role of the school nurse in health education and health promotion in England: a qualitative study

**DOI:** 10.1186/s12912-016-0194-y

**Published:** 2016-12-30

**Authors:** Beverley A. Hoekstra, Vicki L. Young, Charlotte V. Eley, Meredith K. D. Hawking, Cliodna A. M. McNulty

**Affiliations:** Public Health England, Primary Care Unit, Gloucestershire Royal Hospital, Gloucester, GL1 3NN UK

**Keywords:** School nurse, Role, Schools, Health education, Health promotion, Qualitative

## Abstract

**Background:**

The role of the school nurse is complex with many possible elements identified by previous research. The aim of this study is to understand perceptions of the role of the school nurse in order to support school nurses in the delivery of health education.

**Methods:**

The study used an inductive, qualitative research design involving semi-structured interviews and focus groups. Participants were recruited from four NHS trusts across England and final sample size was thirty one school nurses. Three focus groups and two interviews took place in person, and three interviews were over the phone. Data was thematically analysed.

**Results:**

School nurses described six main themes. Four themes directly related to the school nurse role: the main roles of a school nurse, school nurses' role in health education, prioritisation of workload and activities, and community work. A further two other themes related to the delivery of health education: the school nursing system and educational resources.

**Conclusions:**

The role of the school nurse in England is very diverse and the school nurse role in health education is primarily to advise and support schools, rather than to directly deliver education. The study identified that tailored public health educational resources are needed to support school nurses.

## Background

The UK Department of Health states that school nurses have a significant role in co-ordinating and delivering public health interventions for school-aged children [1]. School nurses in England are qualified nurses who hold an additional specialist public health qualification [1]. School nursing assistants are not qualified nurses but are fully and appropriately trained to perform as an assistant to the qualified nurse and are key members of the school nursing team. England has a small school nursing workforce, and the number of school nurses continues to fall [2]. In order to support this limited workforce and their multidisciplinary work, the role of the school nurse in the Healthy Child Programme in England is clearly defined locally [1].

School nurses have been recognised as the leaders in delivering services identified in the Healthy Child Programme from 5 to 19 years [3]. School nurses are key to improving children and young people’s health and wellbeing by delivering health promotion, providing health advice, signposting to other services, active treatment, education, family support, protection, safeguarding, service coordination and multi-agency work [4–8]. While there are many common elements described within the role of school nurses, there is confusion about the school nurse’s exact role [9, 10] and lack of clarification about the role of school nurses in health education and promotion has been well documented [11–15].

This study aims to understand the role of the school nurse from the perspective of the school nurse, using qualitative research methods, in order to better support school nurses in the delivery of health promotion and education.

## Methods

### Sampling and recruitment

Four NHS trusts across England were chosen to include views of school nurses working in different contexts (rural and urban) [Table 1]. School nurses, including school nursing assistants, were recruited through convenience and snowball sampling. Participants were recruited if they were employed as a school nurse or school nurse assistant in the four NHS Trusts involved in this project. In 2014 school nurse leaders were approached by email and telephone, and leaders assisted with the recruitment of study participants which could include school nurse leads, school nurses and school nurse assistants. Snowball sampling was also used to recruit further participants within each area. Final sample size was thirty one school nurses from four English NHS Trusts. Sampling aimed to ensure representation of school nurses working in four different NHS Trusts with different deprivation levels, with a range of experience as a school nurse, and with different numbers of schools they provide care to [Table 2].

**Table 1:** Number of study participants by NHS Trust; 21 school nurses, 2 school nurse leads and 8 school nursing assistants

NHS Trust	*n =* face to face interviews	*n =* telephone interviews	*n =* focus groups	*n =* total
A – in the South West	2	0	0	2
B – in the West Midlands	0	0	5	5
C – in Greater London	0	1	5	6
D – in the East Midlands	0	2	16	18
Total	2	3	26	31

**Table 2:** Participant characteristics who completed the questionnaire

Characteristic	Participants
Role within the School Nurse Team:	
*School Nurse Lead*	2
*School Nurse*	8
*School Nurse Assistant*	5
Years’ experience within a School Nurse Team:	
*0 – 4*	5
*5 – 9*	2
*10+*	8
Number of schools the participant provides care to:	
*0 – 5*	0
*5 – 9*	6
*10+*	7
*No answer*	2
Estimate number of students the participant cares for:	
*0 – 4,999*	2
*5,000 – 9,999*	0
*10,000+*	9
*No answer*	4
Types of schools the participant works with:	
*Primary*	5
*Secondary*	0
*Both*	10

### Ethics

National Research Ethics Committee approval was not required as the study only involved staff. Local approvals were received from each NHS trust involved in the study. The localities have been kept anonymous to maintain participant anonymity. Participants gave written informed consent for participation in the research, audio recording and the publishing of anonymised quotes.

### Interview and focus group topic guide

The interview/focus group topic guide was developed by the lead author (BH), reviewed by the other authors, and piloted with school nurses [Table 3]. The guide integrated aspects of the Theory of Planned Behaviour (TPB), specifically related to school nurses attitudes and barriers to the delivery of health education. The Theory of Planned Behaviour states that the three constructs of personal attitude, subjective norms, and perceived behavioural controls combine to form the intention to carry out a particular behaviour. Understanding barriers to the behaviour of interest would allow the researchers to design training to overcome the barriers using the three constructs of TPB; in this case delivering education around public health issues in schools. Topics covered also included day to day work, changes to the role, health education work, and educational resources including Public Health England’s e-Bug resources covering hygiene and antibiotic resistance topics. See McNulty et al. [16] for a detailed overview of the e-Bug resources. The topic guide in Table 3 was modified as data collection progressed.

**Table 3:** Topic guide for interview and focus groups

Topic Guide	
1) Getting to know you and your role as a school nurse	
Day to day work	
Role of school nurse	
Your role	
Role changed	
Biggest challenges	
Specific guidance documents that influence your work	
2) Health education work	
Health promotion responsibility in schools	
School nurses and microbiology, hygiene, and infections	
Your role in schools relating to health education and health promotion	
Health education you provide	
Assemblies, one on one, or in classroom	
Joint teaching	
Educational resources	
*For those who do not provide education, why not?*	
What do you do that we could help you with?	
Have you considered becoming involved in health promotion or education?	
Does your role change depending on the school you’re in?	
3) e-Bug, and what you and your schools need	
e-Bug topics importance compared to other health protection/promotion activities and teaching	
Impact of educating children about e-Bug topics	
School specific needs for e-Bug topics	
Deliver e-Bug resources	
e-Bug and day-to-day work/workload	
Health resources required	
Tailor resources	
Training	
e-Bug in different languages	
4) Closing questions	
Any further questions	

### Data collection

Semi structured individual interviews and focus groups were facilitated by BH who is an experienced qualitative researcher, registered nurse and researcher for the e-Bug project, Public Health England. Both individual interviews and focus groups were conducted in this research project in order to enrich the data; interviews enabled individuals to speak more openly outside of the group situation, whereas focus groups enabled snowballing and stimulation of ideas. All three focus groups and two interviews took place in person, and three interviews were conducted over the telephone. Interviews and focus groups took place at suitable venues for the participants, i.e. a meeting room in a doctor’s surgery. Two interviews lasted between15-30 min, 3 interviews lasted between 40–50 min and the three focus groups lasted between 45 to 60 min. During the focus groups, a second researcher (VY) was present to record notes, assist BH with collecting consent, and observe the group. Interviews and focus groups were audio recorded, transcribed verbatim by a third party transcription company, and checked for accuracy by BH. No new themes emerged from the later interviews and focus groups at which point data saturation was reached and data sampling was considered complete [17].

Interview and focus group participants were also asked to complete a questionnaire that gathered details about work experience and their role, and half of the participants completed this questionnaire. Characteristics are summarised in Table 2.

### Data analysis

Inductive thematic analysis was conducted based on Braun and Clarke’s (2006) methodology for thematic analysis [18]. NVivo software version 10 was used as a means of organizing and coding data, as well as completing thematic analysis. A coding book was maintained to operationally define each code.

**Fig. 1:**
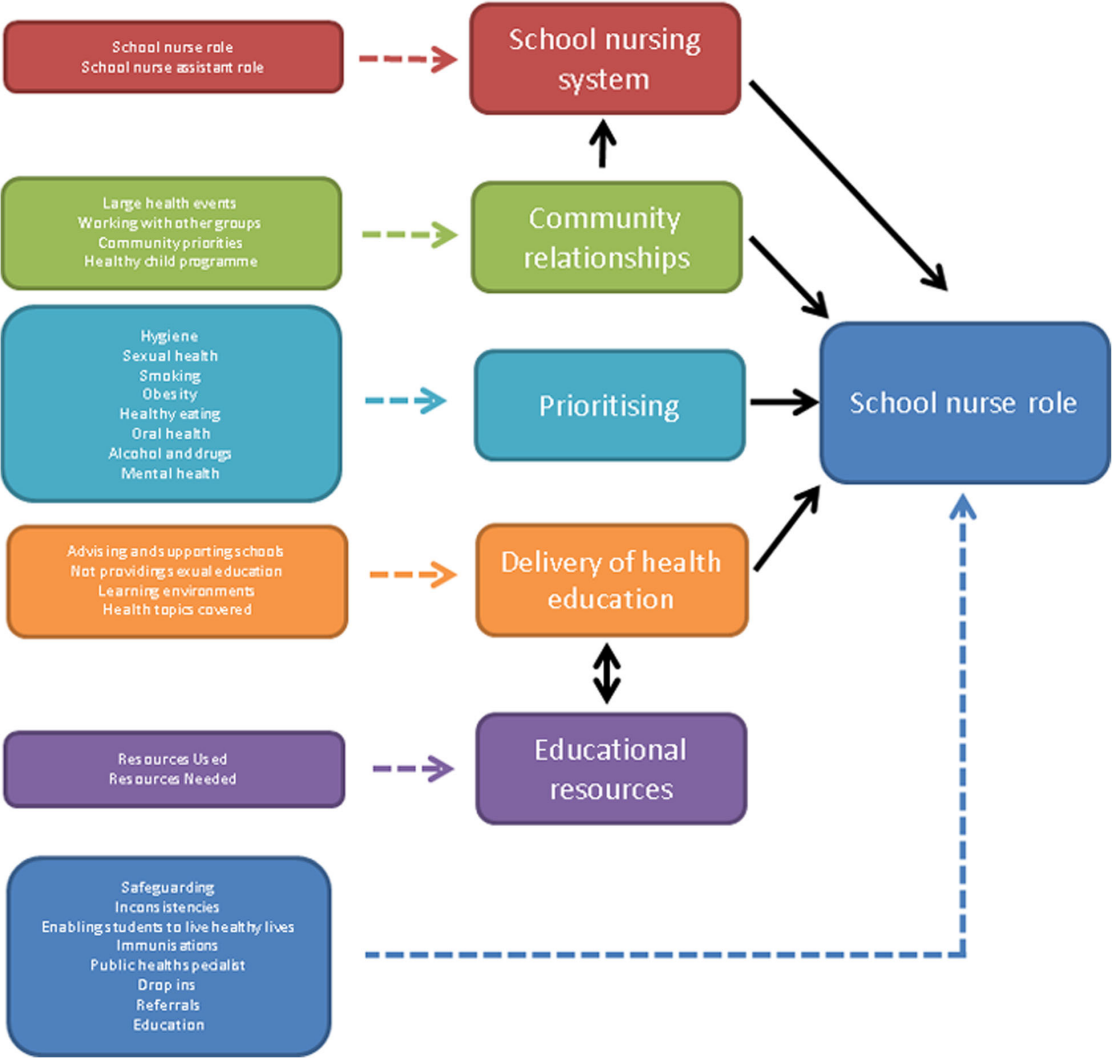
Thematic Framework of the Role of the School Nurse (*black arrow* indicates impactful relationship)

A subset of the data (one focus group and two interviews) were analysed by a second researcher (MH) who is an experienced qualitative researcher. The analysis was discussed and the researchers agreed on emerging themes prior to developing a thematic framework. The framework (Fig. 1) was then finalised in collaboration with the research team. The one sheet of paper method by Ziebland and McPherson (2006) was used to produce a summary report of each theme [19].

## Results

Thirty one school nurses participated from four English NHS Trusts [Table 1]. Half of the participants completed the questionnaire as this questionnaire was developed during data collection and only half the participants had a chance to complete it.

Six overarching themes emerged from the data. Four themes directly related to the school nurse role: the main roles of a school nurse, school nurses'﻿ role in health education, prioritisation of workload and activities, and community work. A further two other themes related to the delivery of health education, and included the school nursing system and educational resources.

### Main roles of a school nurse

School nurses in this study described their work to improve the health of children, young people, families and the community. This included understanding community needs through a local or school health needs assessment, or public health profile; caring for anyone aged 0 to 19 years, though usually 5 to 19 years, regardless of whether they are in school and leading in the delivery of the Healthy Child Programme [3]. School nurses reported that they worked in teams of health care professionals, including qualified school nurses with a public health graduate degree, and school nurse assistants with varying backgrounds and formal training.

The role of school nurses is exceptionally diverse, and school nurses described the service as being *‘stretched’*, and reported being over burdened with work and under resourced. School nurses in this study found it difficult to fit in all they were commissioned to do. At the time of this study, in 2014, safeguarding or child protection [20] was the main element of the role and had increased in recent years leaving less time to contribute to personal, social, health and economic (PSHE) education.

School nurses described that they advised and supported schools with their PSHE education. Some participants, usually school nurse assistants, delivered health education sessions such as assemblies or classroom talks. School nurses signpost schools to resources and services, including specialists such as anaphylaxis or asthma lead, or diabetic nurses. Other aspects of the school nurse role that were identified in this research included running clinics, conducting drop-in sessions, making and responding to referrals, delivering immunisation sessions, supporting students with sexual health needs, conducting health screening sessions including the National Child Measurement Programme [21], supporting parents and working with families, supporting children and young people with long term health conditions (i.e. asthma, epilepsy, leukaemia), and working with schools to meet the needs of students with long term health conditions (i.e. writing care plans).

*“I think, school nurses, I think there is a lot of child protection that takes… precedent, it takes first place. But I think every school nurse, we all, everybody would like to do prevention rather than cure”. SNA1, Rural, Interview*

*“I think safeguarding’s increased, and I, I don’t know the reasons for that, but it’s definitely increased in the last ten years.” SN4, Rural, Focus Group*

*“I think the school nurses are absolutely so resourceful because … we’ve always had big caseloads, we’ve always been short of staff, we’ve always had different challenges. And yet as a group of professionals we manage those so well.” SN7, Rural, Focus Group*

*“But our role is now public health, and as qualified school nurses we attend a public health [course], we do the School Nursing degree, so our view is very much public health and preventative and not just treating the outcomes.” SN1, Urban, Interview*

### School nurses’ role in health education

Participants identified that they have historically played a major role in the delivery of health education in schools, but now the schools themselves are primarily responsible for delivering health education. Some described their main role in health education as supporting teachers, rather than delivering education themselves. School nurses reported delivering education on a range of topics including hygiene, sexual health, mental health and healthy eating in a range of formats such as assemblies and health fairs. Often the amount of health education that school nurses identified they are involved in is based on other commitments and how much capacity they have at certain times. However, school nurses describe that they are always educating, promoting health and sharing key health messages opportunistically.

*“We can advise, and we liaise and support, but we don’t actually carry out the teaching”. SN1, Rural, Interview*

*“That used to be quite a big part of our role, we’d go in and do, yeah lots of health promotion work. But now we don’t have teaching time with anybody at all.” SN1, Rural, Interview*

*“We tend to do, in the high schools and the upper school and colleges, health events rather than…going into the classroom.” SN7, Rural, Focus Group*

*“I think it [health promotion] is very important for them, we’re lucky, I can’t speak for all schools but I know for the schools we have they value what we do in terms of health promotion because sometimes teachers may feel that … they don’t feel confident in that.” SN1, Urban, Interview*

### Prioritisation of workload and activities

School nurses reported that they prioritised their work according to the needs of the young people in their communities. Nurses collaborated with schools to negotiate priorities and develop a school health profile. Some school nurses described that they linked priorities to the broader public health outcomes that contribute to overall public health.

School nurses prioritised tasks daily as they had a very diverse role, were short staffed and their time was limited. A priority for school nurses is supporting schools, especially with the delivery of health education and signposting resources.

Participants reported that priority health education topics included hand hygiene, tooth decay and oral health, sexual health, smoking, mental health, obesity and healthy eating. Some were seasonal, for example hand hygiene was reported as a key priority in the autumn when flu, coughs and colds are widespread.

*“It also depends on the local need; you’ve got to understand your population, what are the issues, what are the public health issues for your area? … One area maybe dental care is an issue, another area maybe it’s not an issue, so you can’t actually put a blanket service for everything because each area and population is different, so it’s got to tailor to the public [in that area]”. SN8, Rural, Focus Group*

*“I think basically we have to prioritise to meet the needs of the young people, so no, depending on how many referrals we get. Safeguarding of course is number one on our priority list, then once we prioritise we see the young people.” SN2, Rural, Focus Group*

*“I think that’s [public health topics] important… especially how germs are spread and colds and flu and it’s very important. “SNA2, Rural, Interview*

*“I think they [public health topics] are essential. I think it’s very important that the message gets across and hopefully for the children then to take it back to the parents”.SNA1, Rural, Interview*

### Community work

School nurses reported undertaking *‘multi-agency work’* and referring children, young people and families to appropriate services and community support (e.g. social care, voluntary sector). School nurses described that they managed stakeholder relationships, supported parents, liaised with schools and maintained good relationships with teachers. Some school nurses indicated that certain schools require nurses to accommodate and be flexible towards the school regarding health education, for example meeting the time and subject requests from schools for health education talks. School nurses in this study identified that good relationships with schools were essential to allow them to access students more easily, as nurses experience barriers when trying to offer school health services. Barriers mentioned included schools prioritising academic lessons or examinations over health services being offered, teachers not having time or interest, and schools not prioritising certain health issues (e.g. sexual health).

*“Sometimes you find that schools often gate keep and don’t want you to get involved”. SN1, Rural, Focus Group*

*“Yeah, yeah. It’s working in partnership really, multiagency working, all the different agencies together to support the family.” SNA1, Rural, Interview*

*“I think they [schools] always welcome somebody from the outside to come in and deliver [health] messages like that.” SN1, Rural, Interview*

*“I think that’s what’s changed…15 years ago…everybody went in and taught contraception, or, or went and taught puberty, or whatever the school identified what they needed. Whereas now we’re looking at the health needs of the local community, looking at that, school as a community and actually the wider community as well, as a whole.” SN8, Rural, Focus Group*

### School nursing system

School nurses identified changes to their role that have taken place in recent years, including changes in team structure, priorities and commissioning. Commissioning is constantly changing, and nurses are only commissioned to offer certain services.

School nurses also identified many challenges they face such as lack of time, lack of capacity, technological barriers and financial barriers. Many nurses involved in this research reported that they could offer a better service if they had more resources in terms of funding, materials and human resources. However, nurses believe they deal with challenges proactively and are very valuable.

*“I think we’re under resourced, I think that’s always been that, hasn’t it?” SN, Rural, Focus Group*

*“Some things we’re commissioned for, some …things like sexual health, like puberty we’re not really [commissioned to do], we can’t support schools with puberty. We can support the teacher and give a little bit of answering questions but we can’t really take the class for puberty [talks]”. SNA2, Rural, Interview*

*“And I think the big challenge is that we’ve been commissioned to provide what’s in that service specification, and it’s that we deliver that and we don’t go delivering things because nobody else is out there to do it, and I think that is a big challenge, because I think you do get pulled in to lots of different things, but it’s making sure that we’re focussed on what we should be doing.”SN1, Urban, Interview*

### Educational resources

School nurses indicated that they are short on educational resources, finances to access resources, and human resources to deliver education. Some school nurses stated they use old or outdated resources, make do with what resources they have, and that they borrow resources from other health professionals (e.g. smoking advisor, dietician). Many nurses mentioned a resource library where they used to access resources, however many of these libraries do not exist anymore (i.e. resources were shared amongst teams when Primary Care Trust’s became Clinical Commissioning Groups in 2013). Most school nurses identified that they make their own resources and tools for teaching. School nurses stated that they are in need of health education resources like the e-Bug resources developed by Public Health England, and some described the need for standardised resources among teams or regions.

*“As I said at the meeting really, we’re very good at making our own resources up. Like the healthy lunchbox, I’ve made myself a healthy and an unhealthy lunchbox up. And we do have books we can get from the British Heart Foundation, places like that, but often it’s collecting bottles of drinks and then putting on how much sugar would be in there. So it is…very much about making our own resources.”SNA1, Rural, Interview*

*“Having a resource where the lesson plan is written out clearly…is good because you need something that is structured, because you’re talking in front of groups of people. With children in school … you need to ensure there’s an aim, there’s an objective, that there’s an evaluation in place at the end of the lesson. So you need something structured and you need the correct resources as well, so that is very important.”SN1, Urban, Interview*

*“Even if we’re not actually delivering, using the resource [e-Bug] ourselves, we’d want to be able to, we could be promoters [of them].”SN, Rural, Focus Group*

*“Probably just support really, and knowing that, even if they were delivering the message, that they’ve got resources they can call upon or people they can call upon, and somewhere that they can actually turn to … if they need that bit of extra support or extra guidance.”SNA1, Urban, Interview*

## Discussion

### Main findings

This research identified that the role of the school nurse in England is very diverse. School nurses’ role in health education has shifted over time from directly delivering health education to advising and supporting schools with health education, as well as signposting resources to teachers. School nurses described that they are in a unique position to have a direct impact on the health and wellbeing of children, young people and their families.

### Comparison with existing literature

As indicated in other previous research [22], we found that there are various elements that make up the school nurse role. The role differed according to location, but this is expected as the needs of young people and communities differ. For example, the needs of children and young people in an urban London community may be very different from the needs of a population in rural Midlands. Given this diversity, it is not surprising that much previous literature [5–10] has identified confusion and misunderstanding about the school nurse role. While our research indicated that the school nurse role is extremely varied and diverse, and school nurses find it difficult to fit in all they are commissioned to do, each school nursing team was fairly clear about their individual role and priorities.

In 2013 the Department of Health identified that there were only 1,200 qualified school nurses in England [23]. In 2012, there were 8.2 million pupils attending 24,372 schools [24]. This equates to 1 school nurse per 6,833 students, or 1 school nurse per 20 schools. The Royal College of Nursing (2015) stated that the number of school nurses in England continue to fall [2], therefore it is not surprising that nurses in this study describe themselves to be under resourced and *‘stretched’* as a profession. Increased time pressures on school nurses are influencing their capacity to directly deliver health education [25]. This research provides further evidence that school nurses have limited time and resources to directly deliver health education alongside their other priorities, and they lead a more advisory and supportive role in health education.

The ‘Our School Nurse’ report by the British Youth Council [26] outlines the views of young people on the role of the school nurse. The views of young people support our research that school nurses are limited in directly delivering health education as the report states that only 5% of young people said that their school nurse has given information on how to have a healthy lifestyle through assemblies or events at school, and only 9% of young people said their school nurse had delivered Sex and Relationship Education or Personal, Social and Health Education lessons [26]. Furthermore, young people state that the best way to learn from the school nursing team is through face-to-face assemblies, presentations and introductory sessions [26] reinforcing the value of school nurses delivering health education topics to young people. Whilst our research suggests that school nurses do not directly deliver health education to schools it is apparent in other literature that young people want to learn from school nurses about health topics.

### Implications for future research

The aim of our research was to identify the school nurse role related to health education in order to better support school nurses, for example by developing tailored educational resources around hygiene and infections. Results show that most school nurses are involved in health education to some degree, whether in a supportive, advisory or signposting role or through directly delivering education. Our research indicates that the amount of health education that school nurses deliver is determined by local priorities and capacity of the school nurse team. Further research could identify the priorities of each locality in England in order to determine the capacity in which school nurses can focus their time on health education.

Through this research, school nurses identified that they are in need of health educational resources to assist in their work. These resources could either be used by school nurses, school nurse assistants, or they could be signposted to schools and teachers by the school nursing teams. Priority resources needed for school nurses include hand hygiene, oral health and sexual health which are currently freely available via the European project e-Bug operated by Public Health England [21, 25]. Resources are also needed to support education about smoking prevention, mental health, obesity and healthy eating. Public Health England’s Change4Life campaign currently provides free toolkits for Key Stage 1 and 2 on healthy eating [25]. School nurses' responsibilities related to health education could be supported by having a bank of evidence based education resources readily available and further research is required to develop additional resources. Signposting free health education resources would allow school nurses to commit more time to their other responsibilities and therefore it would be useful to explore the relationships between school nurses and teachers in sharing good practice.

### Strengths and limitations

A strength of the study was the use of qualitative methods of enquiry to enable comprehensive consideration of the role of the school nurse in the delivery of health education. This study used qualitative methods, interviews and focus groups, which brought synergism, snowballing of ideas and stimulation of participants. However, the convenient nature of the sample could limit transferability of the findings. The topic guide ensured that interviews and focus groups covered similar subjects, and all research was conducted by one researcher (BH) with a background in nursing. This study involved a wide range of school nurses from different educational settings and locations. Data analysis was robust with double coding by an experienced second researcher on a subset of the data, which enhanced the trustworthiness of the findings. In addition, results from our questionnaire [Table 2] indicate that participants had a range of experience, worked in both primary and secondary schools, and were responsible for students in 5 to 10+ schools. A limitation of this aspect of the research, however, is that only half of the study participants completed the study questionnaire.

The findings provide a range of opinions from a diverse group of school nurses, and provide insight into the varied role of the school nurse in England. This work assists in clarifying the role of the school nurse, by those who undertake the role, especially related to health education and promotion.

## Conclusion

The role of the school nurse in England is very diverse and their role in health education is primarily to advise and support schools, rather than to directly deliver health education. The study identified that school nurses can be better supported by having educational resources available to signpost to, enabling school nurses to dedicate more time to their other responsibilities. Addressing understaffing issues would also assist school nurses in their diverse role.

## References

[1] Department of Health. Maximising the school nursing team contribution to the public health of school-aged children. 2014. https://www.gov.uk/government/uploads/system/uploads/attachment_data/file/303769/Service_specifications.pdf Accessed 27 Jul 2016.

[2] Royal College of Nursing. School nurses essential to solve child crisis. 2015. https://www.rcn.org.uk/news-and-events/news/school-nurses-essential-to-solve-child-health-crisis Accessed 27 Jul 2016

[3] Department of Health. Healthy Child Programme: From 5–19 years. 2009. http://www.rcpch.ac.uk/system/files/protected/education/HCP_from-5-19-years-old.pdf Accessed 27 Jul 2016

[4] Department of Health. Getting it right for children, young people and families. 2012. https://www.gov.uk/government/uploads/system/uploads/attachment_data/file/216464/dh_133352.pdf Accessed 27 Jul 2016

[5] Green R, Reffel J (2009). Comparison of Administrators’ and School Nurses’ Perception of the School Nurse Role. J Sch Nurs.

[6] Croghan E, Johnson C, Aveyard P (2004). School nurses: policies, working practices, roles and value perceptions. J Adv Nurs.

[7] Stockman T (2009). Different expectations can lead to confusion about the school nurse’s role. British J School Nursing.

[8] Lightfoot J, Bines W (2000). School nurses: policies, working practices, roles and value perceptions. J Public Health Med.

[9] Whitehead D (2006). The health promoting school: what role for nursing?. J Clin Nurs.

[10] Crabtree E, Davis T (2009). Marketing the role of the school nurse. British J Nursing.

[11] Barnes M, Courtney M, Pratt J, Walsh A (2004). School-based youth health nurses: roles, responsibilities, challenges and rewards. Public Health Nurs.

[12] Bartley J (2004). Health promotion and school nurses: the potential for change. Community Pract.

[13] Cleaver K, Rich A (2005). Sexual health promotion: the barriers school nurse’s face. Community Pract.

[14] Downie J, Chapman R, Orb A, Juliff D (2002). The everyday realities of the multi-dimensional of the high school community nurse. Australian J Advanced Nursing.

[15] Klein J, Sendall M, Fleming M, Lidstone J, Domocol M (2012). School nurses and health education: The classroom experience. Health Educ J.

[16] McNulty C, Lecky D, Farrel D, Kostkova P, Adriaenssens N, Koprivova Herotova T, Holt J, Touboul P, Merakou K, Koncan R, Olczak-Pienkowska A, Britol Avo A, Campos J, on behalf of the e-Bug Working Group (2011). Overview of e-Bug: an antibiotic and hygiene educational resource for schools. J. Antimicrob. Chemother..

[17] Walker J (2012). The use of saturation in qualitative research. Can. J. Cardiovasc. Nurs..

[18] Braun V, Clarke V (2006). Using thematic analysis in psychology. Qual. Res. Psychol..

[19] Ziebland S, McPherson A (2006). Making sense of qualitative data analysis: an introduction with illustrations from DIPEx (personal experiences of health and illness). Med Educ.

[20] Department for Education. The children’s safeguarding performance information framework. 2015. https://www.gov.uk/government/uploads/system/uploads/attachment_data/file/395653/_2015-01-12__The_Childrens_Safeguarding_Performance_Information_Framework.pdf Accessed 27 Jul 2016.

[21] Department of Health. National Child Measurement Programme. 2013. https://www.gov.uk/government/uploads/system/uploads/attachment_data/file/263457/NCMP_schools_guidance__FINAL___2_.pdf Accessed 27 Jul 2016

[22] Brabin L, Stretch R, Roberts S, Elton P, Baxter D, McCann R (2001). The school nurse, the school and HPV vaccination: A qualitative study of factors affecting HPV vaccine uptake. Vaccine.

[23] Department of Health. School nurses to play a bigger role in improving children’s health. 2013. https://www.gov.uk/government/news/school-nurses-to-play-a-bigger-role-in-improving-childrens-health Accessed 27 Jul 2016

[24] Department for Education. Number of schools, teachers and students in England. 2014. https://www.gov.uk/government/publications/number-of-schools-teachers-and-students-in-england/number-of-schools-teachers-and-students-in-england Accessed 27 Jul 2016.

[25] Cotton R (2016). Delivering high quality Personal, Social, Health and Economic (PSHE) education. Br J Nurs.

[26] British Youth Council. Our School Nurse: Young people’s views on the role of the school nurse. 2011. http://www.oxfordhealth.nhs.uk/children-and-young-people/wpcontent/uploads/2014/04/140801-Our-School-Nurse-British-Youth-Council.pdf. Accessed 27 Jul 2016.

